# Horizontal transfer of *vanA* between probiotic *Enterococcus faecium* and *Enterococcus faecalis* in fermented soybean meal and in digestive tract of growing pigs

**DOI:** 10.1186/s40104-019-0341-x

**Published:** 2019-04-12

**Authors:** Ning Li, Haitao Yu, Hongbin Liu, Yuming Wang, Junyan Zhou, Xi Ma, Zheng Wang, Chengtao Sun, Shiyan Qiao

**Affiliations:** 1 State Key Laboratory of Animal Nutrition, College of Animal Science and Technology, China Agricultural University, Beijing, 10093 China; 2 Beijing Key Laboratory of Bio-feed Additives, China Agricultural University, Beijing, 10093 China; 3 National Center for Veterinary Drug Safety Evaluation, College of Veterinary Medicine, China Agricultural University, Beijing, 100193 China

**Keywords:** Chloramphenicol, Digestive tract, Enterococci, Fermented soybean meal, Growing pigs, Probiotics, Transconjugants, *vanA*, Vancomycin

## Abstract

**Background:**

The aim of this study was to investigate the intergeneric transfer of vancomycin resistance gene *vanA* between probiotic enterococci in the fermentation progress of soybean meal and in the digestive tract of growing pigs. One *vanA* genotype vancomycin resistant *E. faecium* strain, E*fm*4, and one chloramphenicol-resistant *E. faecalis* strain, E*fs*2, were isolated from twenty-nine probiotic basis feed material / additive samples. For *in vitro* conjugation, E*fm*4 and E*fs*2 were used as starter to ferment soybean meal. For *in vivo* conjugation, thirty growing pigs were randomly assigned to five groups (*n* = 6), treated with a basic diet, or supplemented with 10% fermented soybean meal, 1% E*fm*4, 5% E*fs*2 or a combination of 1% E*fm*4 + 5% E*fs*2 for 7 d, respectively. Fecal samples of pigs in each group were collected daily for the isolation and dynamic analysis of E*fm*4, E*fs*2 and transconjugants. The sequence types (STs) of E*fm*4, E*fs*2 and transconjugants were analyzed by multilocus sequence typing (MLST). The *vanA* harboring plasmid in E*fm*4 and transconjugants was analyzed by S1-pulsed field gel electrophoresis (PFGE) and further verified by multiple alignments.

**Results:**

The results showed that, in FSBM, transconjugants were detected 1 h after the fermentation, with a conjugation frequency of ~ 10^− 3^ transconjugants / recipient. Transconjugants proliferated with E*fm*4 and E*fs*2 in the first 8 h and maintained steadily for 10 d till the end of the experiment. Additionally, *in vivo* experiment showed that transcojugants were recovered in one of six pigs in both FSBM and E*fm*4 + E*fs*2 groups, with conjugation frequency of ~ 10^− 5^ and ~ 10^− 4^, respectively. MLST revealed the ST of E*fm*4, E*fs*2 and transconjugants was ST1014, ST69 and ST69, respectively. S1-PFGE confirmed the existence of the *vanA*-harboring, 142,988-bp plasmid, which was also a multi-drug resistant plasmid containing *Tn1546*-like transposon.

**Conclusions:**

The findings revealed the potential safety hazard existing in the commercial probiotic enterococci in China, because the horizontal transfer from farm to fork could potentially pose a safety risk to the public.

**Electronic supplementary material:**

The online version of this article (10.1186/s40104-019-0341-x) contains supplementary material, which is available to authorized users.

## Background

Enterococci species are widely distributed in the environment and enable to colonize in the gastrointestinal tract in humans and most animals [1]. For decades, some enterococci strains like *Enterococcus faecium* and *Enterococcus faecalis* are used as probiotics in human and farm animals as a kind of lactic acid bacteria [2]. Previous studies have proven that enterococci can maintain a balance among the intestinal flora [3], promote the absorption of nutrients [4–6] and improve the host immunity [7–9]. In spite of those probiotic properties, however, enterococci have also been known as one of the multiresistant pathogen, ranking among the leading causes of hospital-acquired infections worldwide [10–12].

Vancomycin therapy is one of the most efficient treatments for enterococci infections. While the emergence and rapid spread of vancomycin-resistant enterococci (VRE) represents a particular challenge [13, 14], as there are few remaining therapeutic options for infections caused by VRE [15, 16]. Glycopeptide resistance genes *van* is proven to be the major vancomycin resistant mechanism known in the enterococci. Within all the nine *van* gene types [17], *vanA* is the most frequently indentified one among enterococci isolates from clinical settings [18, 19]. The ability of enterococci to acquire and exchange plasmids and mobile genetic elements that carry antimicrobial resistance gens (like *vanA*) has contributed to their role as multiresistant pathogens within both human and animals.

The spread of VRE as well as *vanA* is not restricted only clinically but they appear also in the community and natural environment as well as animal sectors. For instance, the emergence of VRE in food animal in Europe is proven to have a high association with the use of avoparcin, a glycopeptide antibiotic as growth promoting agent in animal feed [20]. Avoparcin was never approved as a feed additive in China, and worldwide reports on VRE in the livestock industry is limited.

Recently, growing attention has been focused on the worrisome situation of VRE in hospitals or community care centers, which might be traced back to the consumed food, such as yogurt and pork, and eventually to the diet that farm animals intake [21]. Therefore, the aim of this study was to determine the VRE strains in commercial available probiotic enterococci in Beijing, China. Moreover, to assess the potential security issue of these bacteria as an accumulator of antibiotic resistance genes (ARGs), horizontal dissemination of *vanA* between *E. faecium* and *E. faecalis* in fermented soybean meal (FSBM) and the digestive tract of growing pigs were also analyzed.

## Materials and methods

### Bacterial isolates

Enterococci based FSBM and silage corn samples, as well as microbial feed additive samples were purchased in swine farms and local husbandry and veterinary stations in Beijing, China, during June to December, 2016. For FSBM and silage corn samples, enterococci enrichment was performed by adding 25 g sample into 225 mL of Buffered Peptone Water (Beijing Land Bridge, China), vortexed, and then incubated at 37 °C for 24 h. Then 1 mL of the enrichment was added into 9 mL Bile Esculin Azide (BEA; Beijing Land Bridge, China) broth and then incubated 37 °C for 24 h. The changing color of BEA broth from transparent dark brown to opaque black was a sign of the existence of enterococci. A loop of enrichment was streaked on BEA agar plate, incubated 37 °C for 24 h. For feed additive samples, 1 g or 1 mL of the sample was dissolved in 9 mL normal saline, then a loop of the solution was streaked on BEA agar plate, incubated 37 °C for 24 h.

The identification of suspected strains were performed by VITEK 2 Compact Automatic Bacterial Identification and Drug Sensitivity Analysis System (bioMerieux, France) using specific Gram-positive bacteria identification cards (bioMerieux, France), followed by further confirmation of 16S rDNA-based polymerase chain reaction (PCR) screen using universal primers 27-F and 1,492-R, and specific primers for *E. faecium* and *E. faecalis* (Additional file 1: Table S1).

### Antimicrobial susceptibility tests

Isolates confirmed as *E. faecium* or *E. faecalis* were subjected to antimicrobial susceptibility testing to determine their resistance to twenty common antibiotics of nine categories (Additional file 1: Table S2) in accordance with the Clinical and Laboratory Standards Institute (CLSI) [22]. Each isolate was inoculated into 1 mL of sterilized normal saline by picking 3 to 5 colonies using cotton swab from an overnight culture on BHI agar to visually match a McFarland turbidity standard of 0.5. The bacteria liquid was evenly coated onto the surface of un-supplemented Mueller-Hinton (Oxoid, UK) agar plates using cotton swab. Each plate was pasted with five different antimicrobial disks (Beijing Tiantan, China), and then incubated at 37 °C for 24 h. The diameter of inhibition zones was measured to the nearest millimeter and interpreted according to the CLSI standards and previous studies [22, 23]. The *E. faecalis* reference strain ATCC 29212 was used as the quality control.

### Choice of donor and recipient strains

The only VRE strain, *E. faecium* strain, E*fm*4, isolated from a FSBM sample, resistant to vancomycin but susceptible to chloramphenicol, was chosen as donor strain. While another *E. faecalis* strain, E*fs*2, isolated from a microbial feed additive sample, with opposite vancomycin and chloramphenicol resistant phenotype to E*fm*4, was chosen as recipient strain. The genomic DNA of E*fm*4 and E*fs*2 were extracted using Wizard® Genomic DNA Purification Kit (Promega, USA) according to manufacturer’s protocol and further evaluated by PCR for the detection of *van* and *chl* genes. (Additional file 1: Table S1).

Amplified PCR products were purified using Wizard® SV Gel and PCR Clean-Up System (Promega, USA). Cleaned fragments were submitted to Sangon Biotech (Beijing, China) and sequenced from both the forward and reverse stands. The sequence was then analyzed using BLAST.

Freeze-dried powder of E*fm*4 and E*fs*2 were produced by National Feed Engineering Technology Research Center (Beijing, China), with initial bacteria counts ~ 10^10^ CFU/g, respectively, then stored at − 20 °C. Before use, the bacterial activity would be detected by measuring bacterial colony-forming ability after incubation for 24 h at 37 °C on BHI agar plates.

### *In vitro* conjugation: soybean meal fermentation and sample collection

Fresh bacteria liquid cultures of E*fm*4 and E*fs*2 were produced by overnight incubation in BHI broth at 37 °C, respectively, and sub-cultured before use. The initial concentration was adjusted to ~ 10^8^ CFU/mL for both E*fm*4 and E*fs*2. FSBM was conducted in aseptic fermentation bags (5 L, provided by Ministry of Agricultural Feed Industry Centre, Beijing, China) using the following ingredients: sterilized soybean meal (SBM), 500 g; glucose, 20 g; sterilized ddH_2_O, 435 mL; yeast, 5 g; bacteria liquid, 50 mL, with the volume ratio was 1:5 (*v*/*v*, E*fm*4 / E*fs*2) [24, 25]. Two control groups with the same ingredients supplemented with 50 mL E*fm*4 liquid or 50 mL E*fs*2 liquid, respectively, were set.

All fermentation ingredients except E*fm*4 were pre-confirmed to be negative for *vanA*. BHI agar plates containing either 128 mg/L vancomycin or 64 mg/L chloramphenicol were used for detection of E*fm*4 and E*fs*2, respectively. BHI agar plates supplemented with vancomycin and chloramphenicol were used for the detection of transconjugants. Five replicates were maintained for each treatment. E*fm*4, E*fs*2 and transconjugants were counted through serial plating on selective BHI agar plates at 0, 1, 2, 4, 6, 8, 12, and 24 h, and then daily from day 2 to day 10.

On each selective plate, five clones were randomly selected, cultured, identified by species assessment as described above. Putative transconjugants obtained on double resistant selective plates were further confirmed by PCR amplification on the detection of *van* and *chl* genes [26, 27].

The conjugant frequency was calculated using the equation described by Liu et al. [28]:

$$ \left(\mathrm{Conjugation}\right)\ \mathrm{frequency}=\frac{\mathrm{Clones}\ \mathrm{of}\ \mathrm{Transconjugants}\ \left(\mathrm{CFU}/\mathrm{plate}\right)}{\mathrm{Clones}\ \mathrm{of}\ \mathrm{Recipients}\ \left(\mathrm{CFU}/\mathrm{plate}\right)} $$

### *In vivo* conjugation: animals, feeding and sample collection

All procedures used in these experiments were conducted in accordance with the Chinese Guidelines for Animal Welfare and were approved by the China Agricultural University Institutional Animal Care and Use Committee (Beijing, China) [29]. The pig cages, pigpens and feeding appliances were pre-disinfected, then confirmed the absence of enterococci as well as *van* and *chl* genes using methods described previously [30].

A total of thirty barrows (Duroc × Landrace × Yorkshire) at the age of 60 d, with initial body weight of 15.38 ± 2.8 kg were individually housed and provided with a non-medicated corn-soybean meal basal diet and sterilized water *ad libitum* for 10 d. Fresh fecal samples were collected daily to confirm the absence VRE and *vanA*. After acclimatization, pigs were randomly assigned to one control group (*n* = 6), fed with the same basal diet previously described; and four treatment groups (*n* = 6), fed with basal diet supplemented with 10% FSBM, 1% E*fm*4, 5% E*fs*2 or a combination of 1% E*fm*4 + 5% E*fs*2 for 7 d, respectively.

The estimation of FSBM consumption was about 5 kg/d, so 5 bags of FSBM were performed daily, from − 1 d to 6 d of the experiment as described above. The fermentation process lasted for 2 d. At this time point, the bacteria concentrate of E*fm*4 and E*fs*2 was both ~ 10^9^ CFU/g FSBM. Then 5 kg fresh FSBM were uniformly dispersed into 45 kg basal diet to feed FSBM group barrows. Meanwhile, the initial bacteria concentrate of E*fm*4 in E*fm*4 group and E*fm*4 + E*fs*2 group was ~ 1.0 × 10^8^ CFU/g feed, and the initial bacteria concentrate of E*fs*2 in E*fs*2 group and E*fm*4 + E*fs*2 group was ~ 5.0 × 10^8^ CFU/g feed, respectively.

Fresh rectal feces sample of each pig was collected once every 24 h for serial plating and counting of E*fm*4, E*fs*2, and transconjugants using selective BHI agar plates. Donor, recipient and transconjugant strains on selective plates and then resistant genes were identified as described above.

### Multilocus sequence typing

To investigate the genetic heterogeneity of the donor, recipient and transconjugants, the MLST analysis was performed. 7 housekeeping genes were amplified using the primers and protocol specified by MLST website (https://pubmlst.org/efaecium/ and https://pubmlst.org/efaecalis/), respectively. Allele and sequence types (STs) assignments were made at the publicly accessible MLST database described above [2, 31].

### S1-PFGE and southern hybridization

S1-PFGE was performed as described previously [2]. Overnight incubated cells of E*fm*4, E*fs*2 and transconjugants were embedded in InCert Agarose (Lonza, USA), respectively, followed by digestion with S1 nuclease (TaKaRa, Japan). The DNA restriction fragment were separated in 1% SeaKem Gold® Agarose (Lonza, USA) using a pulse gel electrophoresis apparatus (Bio-Rad, USA). Genomic DNA of *Salmonella serovar* Braenderup strain H9812, digested with *XbaI* (TaKaRa, Japan), was used as a molecular standard.

Southern hybridization was performed on the S1-PFGE gel using DIG High Prime DNA Labeling and Detection Starter Kit II (Roche, Germany) with digoxin- labeled DNA probes specific for *vanA*, followed by manufacturer’s protocol.

### Plasmid sequencing

The whole genome of E*fm*4 including *vanA*-harboring plasmid was sequenced using the Pacific Biosciences RS II (Pacific Biosciences, Menlo Park, CA, USA) sequencing platform. *De novo* assemblies of Pacbio Reads were performed by SMRT Analysis pipeline v2.3.0 in conjunction with RS_HGAP_Assembly. Three protocols and additional assemblies were performed by minimus 2 from the AMOS package [32]. The open reading frames (ORFs) were identified and annotated with the prodigal.

The *vanA*-carrying plasmids from transconjugants obtained from FSBM and E*fm*4 + E*fs*2 group were partially sequenced (~ 13,000 bp) to verify their transfer into E*fs*2. By targeting the regions adjacent *vanA*, primers (Additional file 2: Supplemental data 1) were designed based on the sequence of *vanA*-carrying plasmid in E*fm*4. Multiple alignment of acquire sequence was conducted using SnapGene (Version 4.2.6) against the sequence of *vanA*-carrying plasmid E*fm*4.

## Results

### Antimicrobial susceptibility

Among twenty-nine enterococci isolates, two strains (6.90%, 2/29) were susceptible to all the twenty antibiotics tested; the remaining strains (93.10%, 27/29) showing resistance to at least one antibiotic (Table 1). An *E. faecium* strain, E*fm*4, exhibited a high MIC to vancomycin (> 1,024 μg/mL) harboring *vanA*, while an *E. faecalis* strain, E*fs*2, resistant to chloramphenicol (128 μg/mL) carrying *catA1* on genome were selected as donor and recipient strains (Table 2).

**Table 1:** Antimicrobial susceptibilities and sequence types of enterococci isolated from feed/feed additives

Strain	Origin	Antibiotics																				ST
		VA	TCL	RA	C	AM	PIP	CZ	P	MPN	AMX	OFL	CIP	GTF	GM	TE	MNO	E	KIA	FT	FU	
E*fm*1	FSBM	\	\	\	\	\	\	R	\	\	\	\	\	\	\	\	\	\	\	\	R	ST94
E*fm*2	FSBM	\	\	\	\	\	\	\	\	\	\	\	\	\	\	\	\	\	\	\	\	ST40
E*fm*3	FSBM	\	\	\	\	\	\	\	\	\	\	\	\	\	\	\	\	\	\	\	\	ST296
E*fm*4	FSBM	R	R	\	\	R	R	R	R	R	R	R	R	R	R	\	\	R	R	\	R	ST1014
E*fm*5	FSBM	\	\	I	\	\	\	\	\	\	\	\	\	\	\	\	\	I	\	\	R	ST 296
E*fm*6	FSBM	\	\	\	\	\	\	R	\	\	\	\	\	\	\	\	\	I	\	\	R	ST 94
E*fm*7	FSBM	\	\	\	\	\	\	\	\	\	\	\	\	\	\	\	\	I	\	\	R	ST 6
E*fm*8	FSBM	\	\	I	\	\	\	\	\	\	\	\	\	\	\	\	\	\	\	\	R	ST 5
E*fm*9	FSBM	\	\	\	\	\	\	\	\	\	\	\	\	\	\	\	\	\	\	\	R	ST 60
E*fm*10	FSBM	\	\	\	\	\	\	R	\	\	\	\	\	\	\	\	\	\	\	\	R	ST 6
E*fm*11	Silage corn	\	\	\	\	\	\	R	\	R	\	\	\	\	\	\	\	I	\	\	R	ST 812
E*fm*12	Silage corn	\	\	I	\	\	\	\	\	\	\	\	\	\	\	\	\	\	\	\	R	ST 5
E*fm*13	Silage corn	\	\	I	\	\	\	\	\	\	\	\	\	\	\	\	\	I	\	\	R	ST 5
E*fm*14	Silage corn	\	\	I	\	\	\	\	\	\	\	\	\	\	\	\	\	I	\	\	R	ST 178
E*fm*15	Silage corn	\	\	\	\	\	\	\	\	\	\	\	\	\	\	\	\	\	\	\	R	ST 160
E*fm*16	Silage corn	\	\	I	\	\	\	\	\	\	\	\	\	\	\	\	\	R	R	\	R	ST 726
E*fm*17	Feed additive	\	\	\	\	\	\	R	\	\	\	\	\	\	\	\	\	I	\	\	R	ST812
E*fm*18	Feed additive	\	\	I	\	\	\	R	\	\	\	\	\	\	\	\	\	I	\	\	R	ST 5
E*fm*19	Feed additive	\	\	I	\	\	\	\	\	\	\	\	\	\	\	\	\	I	\	\	R	ST 5
E*fm*20	Feed additive	\	\	\	\	\	\	\	\	\	\	\	\	\	\	\	\	\	\	\	R	ST 5
E*fm*21	Feed additive	\	\	I	\	\	\	\	\	\	\	\	\	\	\	\	\	I	\	\	R	ST 361
E*fm*22	Feed additive	\	\	R	\	\	\	\	\	\	\	\	\	\	\	\	\	\	\	\	R	ST 21
E*fm*23	Feed additive	\	\	I	\	\	\	\	\	\	\	\	\	\	\	\	\	\	\	\	R	ST 21
E*fm*24	Feed additive	\	\	\	\	\	\	\	\	I	\	\	\	\	\	\	\	\	\	\	R	ST 695
E*fm*25	Feed additive	\	\	\	\	\	\	I	\	\	\	\	\	\	\	\	\	I	\	\	R	ST 24
E*fm*26	Feed additive	\	\	\	\	\	\	\	\	\	\	\	\	\	\	\	\	I	\	\	R	ST 24
E*fm*27	Feed additive	\	\	I	\	\	\	\	\	\	\	\	\	\	\	\	\	\	\	\	R	ST 21
E*fs*1	Feed additive	\	\	\	\	\	\	I	\	\	\	\	\	\	\	\	\	I	\	\	R	ST 69
E*fs*2	Feed additive	\	\	\	R	\	\	\	\	\	\	R	R	I	R	R	\	R	R	\	\	ST 69

E*fm*, *E. faecium* isolates;

E*fs*, *E. faecalis* isolates;

\, susceptible;

R, resistant;

I, intermediate;

ST, sequence type;

VA, Vacomycin; TCL, Teicoplanin; RA, Rifampicin; C, Chloramphenicol; AM, Ampicillin; PIP, Piperacillin; CZ, Cefamedin; P, Penicillin; MPN, Meropenem; AMX, Amoxicillin; OFL, Ofloxacin; CIP, Ciprofloxacin; GTF, Gatifloxacin; GM, Gentamicin; TE, Tetracycline; MNO,

Minocycline; E, Erythromycin; GI, Kitasamycin; FT, Nitrofurantoin; FU, Furazolidone

**Table 2:** Antimicrobial susceptibilities and sequence type of donor strain E*fm*4 and recipient strain E*fs*2

Strain	Origin	Antibiotics																				ST
		VA	TCL	RA	C	AM	PIP	CZ	P	MPN	AMX	OFL	CIP	GTF	GM	TE	MNO	E	KIA	FT	FU	
E*fm*4	FSBM	R	R	\	\	R	R	R	R	R	R	R	R	R	R	\	\	R	R	\	R	ST1014
E*fs*2	Feed additive	\	\	\	R	\	\	\	\	\	\	R	R	I	R	R	\	R	R	\	\	ST69

E*fm*, *E. faecium* isolates;

E*fs*, *E. faecalis* isolates;

\, susceptible;

R, resistant;

I, intermediate;

ST, sequence type

VA, Vacomycin; TCL, Teicoplanin; RA, Rifampicin; C, Chloramphenicol; AM, Ampicillin; PIP, Piperacillin; CZ, Cefamedin; P, Penicillin; MPN, Meropenem; AMX, Amoxicillin; OFL, Ofloxacin; CIP, Ciprofloxacin; GTF, Gatifloxacin; GM, Gentamicin; TE, Tetracycline; MNO,

Minocycline; E, Erythromycin; GI, Kitasamycin; FT, Nitrofurantoin; FU, Furazolidone

### *In vitro* conjugation

**Fig. 1:**
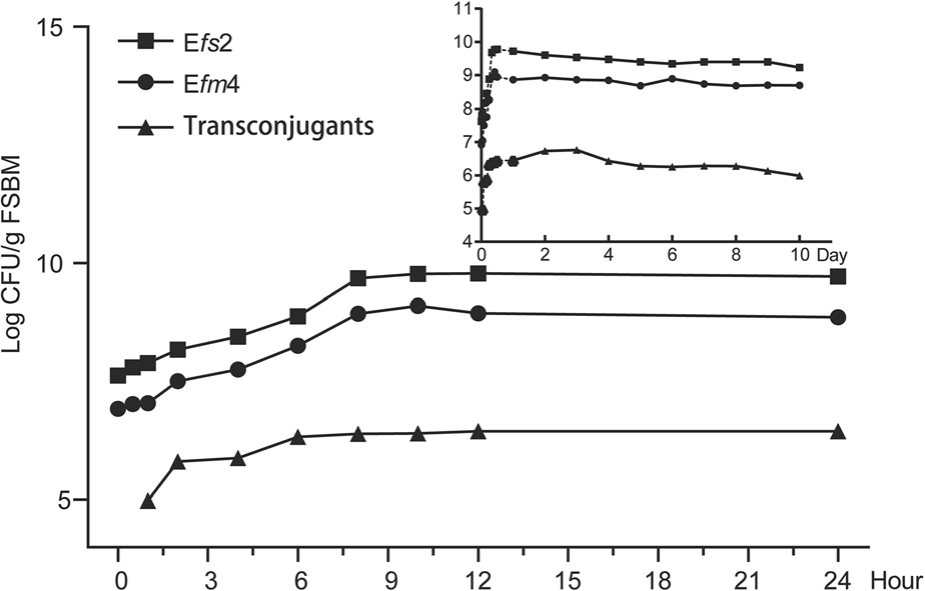
Growth kinetics of E*fm*4, E*fs*2 and transconjugants during the fermentation process of soybean meal. The solid circular represents the growth kinetics of the donor strain E*fm*4. The solid square represents the growth kinetics of the donor strain E*fm*4; the solid minute triangle represents the growth kinetics of the transconjugants. E*fm*4, *vanA* positive strain *Enterococcus faecium* E*fm*4; E*fs*2, chloramphenicol-resistant strain *Enterococcus faecalis* E*fs*2

During the fermentation process, the donor strain E*fm*4 and the recipient strain E*fs*2 both grew well and had similar growth kinetics. Concentrations of E*fm*4 reached a maximum of 1.25 × 10^9^ CFU/g FSBM at hour 10, with the concentrations of E*fs*2 reached a maximum of 6.0 × 10^8^ CFU/g FSBM as well (Fig. 1). Transconjugants were detected at the first detection point of fermentation (1 h), with the cell count at 0.94 × 10^5^ CFU/g FSBM, and the conjugation frequency being 1.2 × 10^− 3^ (0.94 × 10^5^ CFU/g FSBM / 7.8 × 10^7^ CFU/g FSBM, transconjugants/E*fs*2). The number of transconjugants increased continuously and then peaked at day 3, at 5.8 ×  10^6^ CFU/g FSBM. Moreover, the transconjugants remained consistently at ~ 10^6^ CFU/g FSBM during day 1 (from hour 6) to day 10 (Fig. 1).

### *In vivo* conjugation

No pigs were colonized by vancomycin or chloramphenicol-resistant enterococci prior to the study (day−10 to − 1, data not shown). Besides, during the test period, no VRE strain was detected in the fecal samples collected from the control group fed with basil diet.

**Fig. 2:**
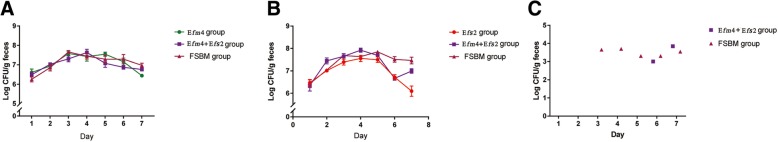
Viable count of E*fm*4, E*fs*2 and transconjugants in feceal samples of growing pigs. (**a**): The growth kinetics of donor strain E*fm*4 in E*fm*4 group, E*fm*4+ E*fs*2 group and FSBM group; (**b**): The growth kinetics of receiver strain E*fs*2 in E*fs*2 group, E*fm*4+ E*fs*2 group and FSBM group; (**c**): The growth kinetics of transconjugants in E*fm*4+ E*fs*2 group and FSBM group. In both E*fm*4+ E*fs*2 group and FSBM group, the transconjugants were only detected in one pig out of six. Results from fecal samples obtained at 24 h before the experiment are plotted as day zero. FSBM, fermented soybean meal; E*fm*4, *vanA* positive strain *Enterococcus faecium* E*fm*4; E*fs*2, chloramphenicol-resistant strain *Enterococcus faecalis* E*fs*2

The donor, recipient, and transconjugants exhibited similar growth kinetics, indicating that these two strains could stably reside the digestive tract of growing pigs, and therefore there was no growth defect that could have affected the results (Fig. 2a and b). In the fecal samples obtained from E*fm*4 + E*fs*2 group, the concentration of E*fm*4 and E*fs*2 reached a maximum of 6.44 × 10^7^ CFU/g fecal and 9.28 × 10^7^ CFU/g fecal at day 4, respectively (Fig. 2a). Similarly, the concentrations of E*fm*4 and E*fs*2 peaked at 4.88 × 10^7^ CFU/g and 7.28 × 10^7^ CFU/g on day 3 and day 5 in the FSBM group, respectively (Fig. 2b). Notably, transconjugants were observed in both two groups, although only positively detected in one pig of six for each group. In E*fm*4 + E*fs*2 group, the conjugation frequency reached to 1.97 × 10^− 4^ and 6.25 × 10^− 4^ at day 6 and day 7 (Fig. 2c, Table 3), respectively. In contrast, the transconjugant was more frequently occurred, with a conjugation frequency of 9.4 × 10^− 5^, 4.60 × 10^− 4^, 2.75 × 10^− 5^, 5.0 × 10^− 5^ and 9.4 × 10^− 5^, from day 3 to day 7, respectively (Fig. 2b, Table 3).

**Table 3:** Colony count of E*fs*2 and transconjugants from fecal samples of *in vivo *conjugation

Day		E*fs*2, × 10^6^ CFU/g fecal						Transconjugants, × 10^3^ CFU/g fecal					
1	E*fs*2	7.60	1.50	0.80	4.50	4.90	3.30						
	E*fm*4 + E*fs*2	3.70	0.20	7.50	8.70	1.10	2.30	0	0	0	0	0	0
	FSBM	0.55	2.85	4.90	5.50	1.35	6.50	0	0	0	0	0	0
2	E*fs*2	12.50	8.20	17.50	8.85	11.25	6.30						
	E*fm*4 + E*fs*2	15.00	16.50	27.00	14.50	55.00	86.00	0	0	0	0	0	0
	FSBM	12.30	15.00	11.30	12.50	7.50	8.50	0	0	0	0	0	0
3	E*fs*2	22.50	8.50	13.50	55.00	36.00	42.00						
	E*fm*4 + E*fs*2	33.00	42.00	47.50	14.50	73.00	107.00	0	0	0	0	0	0
	FSBM	41.00	47.5	36.00	46.00	58.00	59.50	0	0	0	4.50	0	0
4	E*fs*2	68.00	77.00	34.50	55.50	36.00	57.00						
	E*fm*4 + E*fs*2	175.00	88.00	114.00	39.50	55.00	85.00	0	0	0	0	0	0
	FSBM	20.50	19.00	21.00	6.00	12.20	3.50	0	0	0	5.00	0	0
5	E*fs*2	55.00	101.5	23.00	27.00	72.00	36.00						
	E*fm*4 + E*fs*2	65.00	51.00	41.00	35.5	65.00	4.00	0	0	0	0	0	0
	FSBM	43.00	65.00	87.00	95.00	86.5	60.00	0	0	0	2.00	0	0
6	E*fs*2	3.50	1.85	5.30	9.40	7.80	8.30						
	E*fm*4 + E*fs*2	3.75	5.80	9.50	4.60	4.50	2.30	0	1.00	0	0	0	0
	FSBM	55.00	18.00	14.00	34.00	27.00	92.00	0	0	0	2.00	0	0
7	E*fs*2	6.55	7.80	1.55	1.80	3.80	6.75						
	E*fm*4 + E*fs*2	6.70	12.10	18.50	5.50	18.50	6.00	0	7.00	0	0	0	0
	FSBM	10.00	12.00	27.0	46.50	84.00	45.00	0	0	0	3.50	0	0

E*fm*, *E. faecium* isolates;

E*fs*, *E. faecalis* isolates;

FSBM, fermented soybean meal

The presence *vanA* and *catA1* in transconjugants was confirmed by further PCR bothly* in vitro* and *in vivo* experiments.

### Molecular typing of probiotic enterococci isolates

The MLST analysis identified that the ST of E*fm*4, E*fs*2 and transconjugants was ST1014, ST69 and ST69, respectively (Table 1).

### S1-PFGE and southern hybridization

**Fig. 3:**
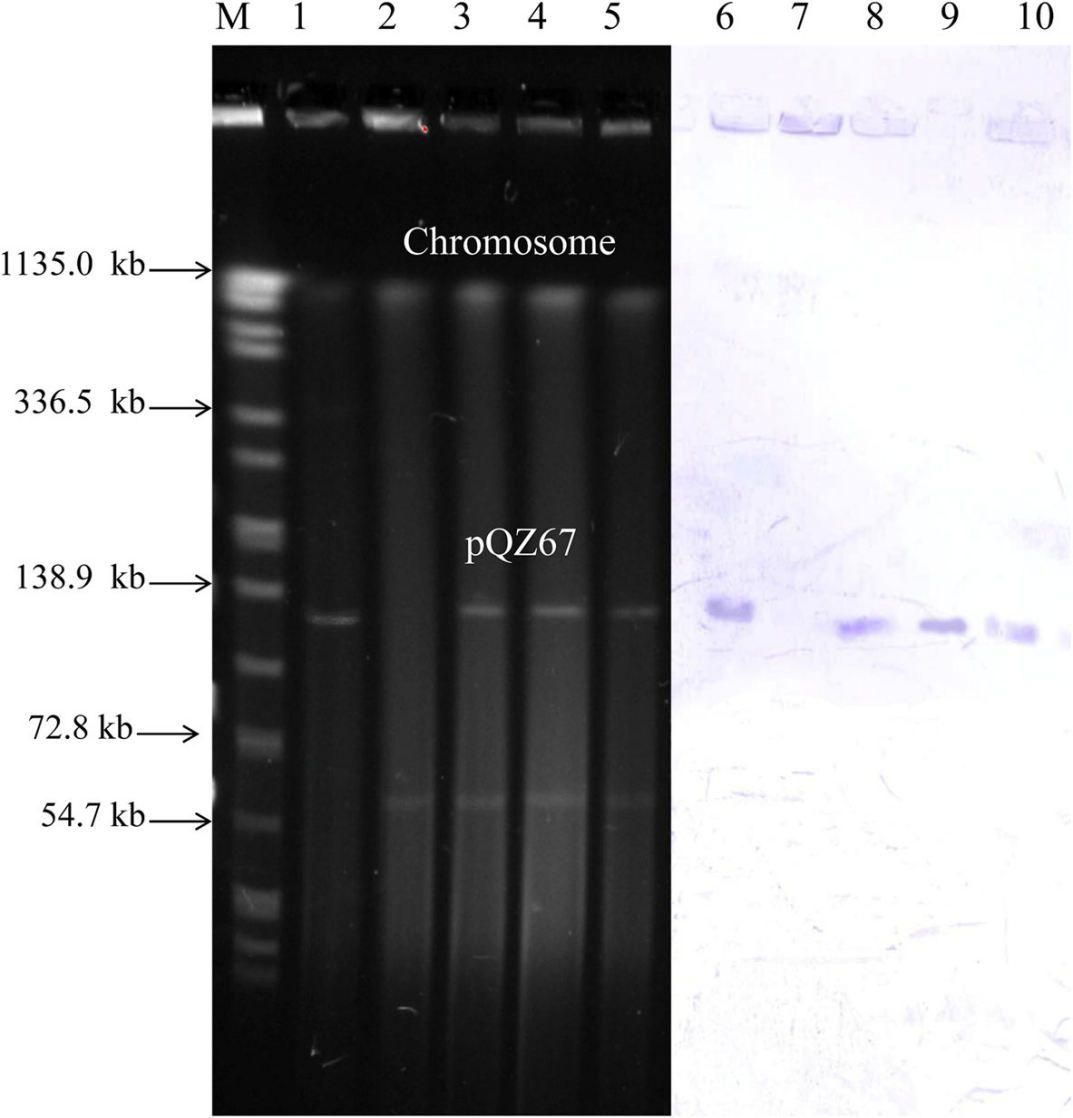
S1-PFGE analysis of E*fm*4, E*fs*2 and transconjutions and southern hybridization. Line M, molecular weight marker *Salmonella braenderup* H9812; Line 1 and 6, donor strain *E. faecium* E*fm*4; Line 2 and 7, recipient strain *E. faecalis* E*fs*2; Line 3 and 8, transconjugants obtained from FSBM; Line 4 and 9, transconjugants obtained from E*fm*4+ E*fs*2 group; Line 5 and 10, transconjugants obtained from FSBM group. FSBM, fermented soybean meal; PFGE, pulsed-field gel electrophoresis; E*fm*4, vanA positive strain *Enterococcus faecium* E*fm*4; E*fs*2, chloramphenicol-resistant strain *Enterococcus faecalis* E*fs*2

S1-PFGE analysis exhibited one visible plasmid bands (~ 140 kb) in the donor strain E*fm*4 and another bands (~ 60 kb) in the recipient strain E*fs*2. It could be observed clearly that the transconjugants harbored two visible plasmids from both E*fm*4 and E*fs*2 (Fig. 3).

Southern hybridization confirmed that *vanA* hybridized on the visible plasmid of E*fm*4 with a size of ~ 140 kb, which was subsequently named pQZ67 in this study.

### Analysis of the plasmid pQZ67

**Fig. 4:**
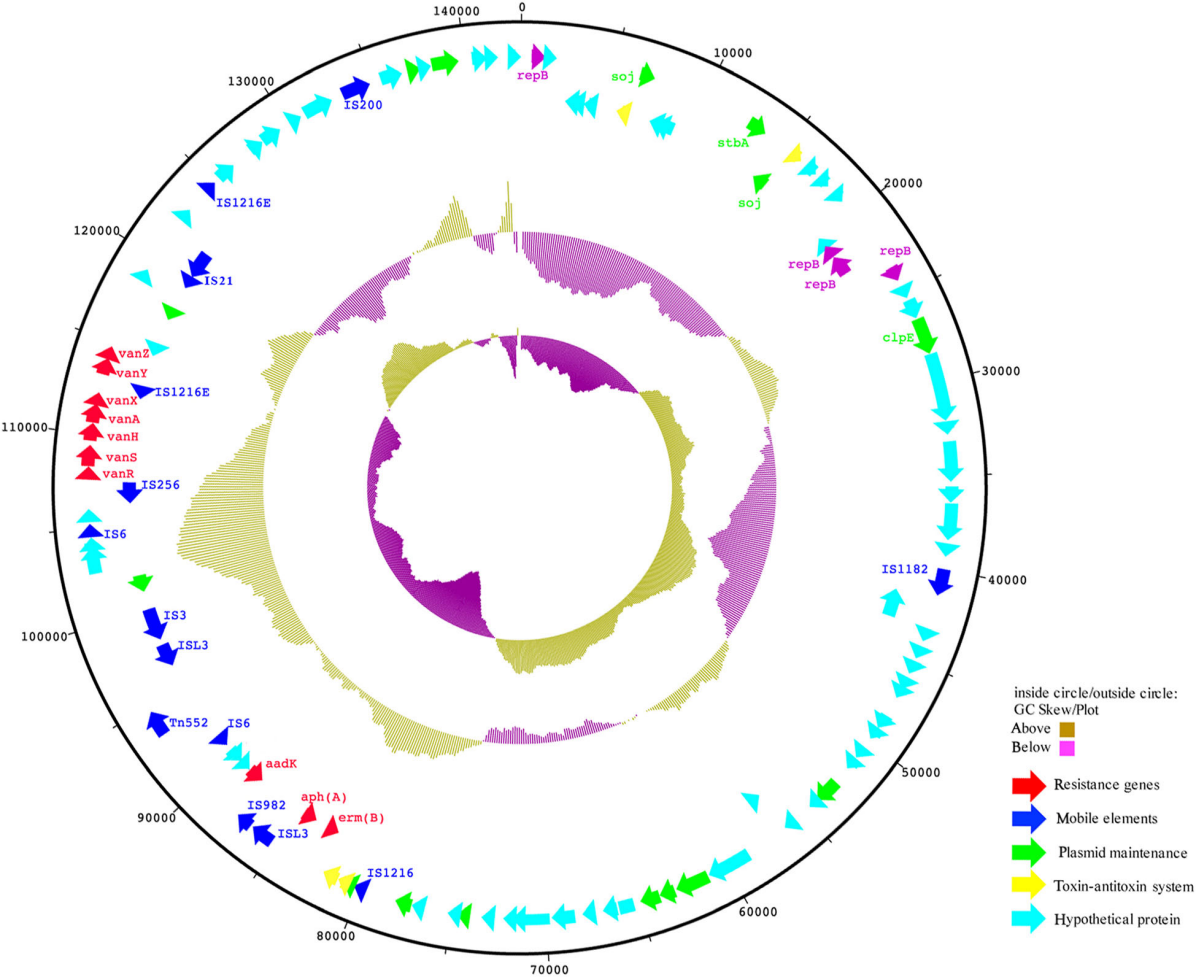
Genetic map of the *vanA* harboring plasmid pQZ67. The circles display (from the outside to inside): (**i**) size in bp; (**ii**) positions of the predicted coding sequences transcribed in clockwise orientation; (**iii**) position of predicted coding sequences transcribed in counterclockwise orientation; (**iv**) GC content plotted against 50%, with brown indicating> 50% and purple indicating< 50%; and (**v**) GC skew [(G + C)/(G-C)] in a window of 300 bp

The complete DNA sequence of plasmid pQZ67 was obtained by whole-genome sequencing (Fig. 4). 142,988-bp pQZ67 consisted of 38,000 amino acid residues and containing 185 potential edcoding sequences (CDSs), with a G + C content of 34.03%. The *vanRSHAX* gene cluster was located between two insertion sequences which were IS*21*and IS*256*. It was a *Tn1546*-like compound transposon, which played a crucial role in horizontal transmission and might assist in the horizontal transfer of *vanA* in Enterococcaceae.

Multiple alignment showing the *vanA*-carrying fragments (~ 13,780 bp) from the FSBM as well as E*fm*4 + E*fs*2 group were identical to the corresponding region of *vanA*-carrying plasmid in E*fm*4, expect single base mutation within *vanA* gene in transconjugants (Additional file 2: Supplemental data 1).

## Discussion

To the best of our knowledge, this is the first time of the observation on the transfer of *vanA* of probiotic origin occurred in the process of FSBM and in the digestive tract of growing pigs. Over the past few decades, with the extensive emergence of antibiotic-resistant strains, probiotics are increasingly used in human and food animals as suitable replacements of antibiotics [33, 34]. As traditional lactic acid bacteria, *E. faecium* and *E. faecalis* have been wildly used in foods, drugs, dietary supplements, microbial feed and feed additives [35]. However, some prospective studies showed that these bacteria can serve as an accumulator of antibiotic resistance genes (ARGs) potentially providing them to pathogens, raising the problem of pathogen resistance [36, 37]. For the past two decades, the production and consumption of microbial products associated with the increase in intensive livestock farming, and the safety of these products is important for food safety. Previous studies have shown concern on probiotics applied in feed containing transferable ARGs [38, 39]. Using filter mating experiments, Nawaz et al. [40] found that *ermB* and *tetM* from lactobacillus strains could be successfully transferred to *E. faecalis*, with conjugation frequency ranged from 10^− 5^ to 10^− 6^. Moreover, Ma et al. [41] used *Lactobacillus delbrueckii* strain harboring *mcrC* as donor strain, and *E. faecalis* ATCC 29212 as recipient strain, resulting in a conjugation frequency as high as 2.2 × 10^− 2^. Similarly, our study found out that, during the production of FSBM, the *vanA* could be transferred easily at the early stage (detected only 1 h after the fermentation began), and the transconjungations increased steadily during the whole fermentation process of FSBM. Nevertheless, the conjugation frequency ranged from ~ 10^− 3^ to ~ 10^− 4^, which was about 10 to 100 fold higher than primary studies [42, 43]. It might be because that FSBM provided a suitable environment for conjugations to happen, where three necessary conditions were need: full contact, absolute stationary and sufficient nutrition [44, 45].

Most of the previous experiments of resistance genes transfer *in vivo* were performed on specific pathogen free (SPF) or gnotobiotic animals. Bourgeois- Nicolaos et al. [46] revealed that *vanA* could transfer from pig derived *E. faecium* to human derived *E. faecalis* in germ-free mice intestines, but only 2 colonies of transconjugants were detected after administration donor and receiver strains intragastrically for 14 d. Moreover, the transfer of *vanA*-harboring plasmid from poultry and pig derived strain to human fecal *E. faecium* strains in digestive tract of germ-free mice was observed by Dahl et al. [47], indicating that even transient colonization strains might provide a significant reservoir to transfer resistance genes to permanent commensal bacteria. A study by Lester et al. [48] demonstrated that conjugation could occur in the human gastrointestinal tract. In this study, three of six volunteers who ingested ~ 10^9^ CFU *vanA*-harboring *E. faecium* bacteria in 250 mL milk had transconjugant bacteria in their feces at a mean conjugation frequency of ~ 10^− 7^ transconjugants / recipient. This was in line with the observations in our results, in particular for the E*fm*4 + E*fs*2 group, as the transconjugants were obtained 5 d after ingesting E*fm*4 together with E*fs*2, and the conjugation frequency was ~ 10^− 4^ to ~ 10^− 5^.

It was noteworthy that transconjugants in the FSBM passed through the digestive tract of growing pigs successfully and could be detected after 3 d of feeding. Moreover, this cycle was 2 d sooner than feeding E*fm*4 and E*fs*2 directly. In addition, in commercial production facility, the SBM fermentation process usually lasted only for 2 to 5 d before further processing, and the inoculation amount of bacteria was much more than we used in the current study [49]. This emphasized the possibility that the resistance genes harbored by commercial probiotics used in feed and feed additives can be disseminated in digestive tracts of animal, which may cause further infections under extreme conditions.

Many researchers explored the transferability of resistance genes from farm to fork [50, 51]. Nawaz et al. [40] analyzed the antibiotic resistance in lactic acid bacteria from retail fermented foods in Xi’an, China. Not only did they identify *tetS* gene from *Lactobacillus brevis* and *Lactobacillus kefiri* for the first time, but they also observe the *ermB* gene from *L. fermentum* and *L. salivarius*. Furthermore, the *tetM* gene from *L. plantarum* and *L. brevis* was also observed to be successfully transferred to *E. faecalis* by filter mating, with a conjugation frequency as ~ 10^− 6^ to ~ 10^− 5^. In addition, He et al. [52] analyzed the oxazolidinone / phenicol resistance gene *optrA* from 17 non-related *E. faecalis* isolates from human and animal origin, and the IS1216E elements on the plasmid were also found to contribute the most in the dissemination of the *optrA* gene. The IS 1216E elements were also detected on pQZ67 plasmid in our study, demonstrating the likelihood of *in vitro* and *in vivo* transfer of *vanA* between enterococci and other Gram-positive bacteria from food or feed to animal or human via food chain *in vitro* and *in vivo*.

In this study, the diversity of STs showed that the commercial probiotic enterococci strains were from multiple sources. Additionally, it should be noted that the sequence type of VRE strain E*fm*4 was ST1014 (Fig. 3). ST1014 was evolved out of ST78, which was the dominant clone complex (CC) in most cities in China, causing the prevalence and spread of VRE [53]. Furthermore, another reported vancomycin resistant *E. faecium* strain with the same ST1014 was isolated in a hospital in Shandong Province, China, in 2013 [53]. Although there was no evidence showing the direct link between that isolate and E*fm*4, the possible affiliation between ST1014 and ST78 (CC17) still rang the alarms of the safety of probiotic enterococci applied in feed and food.

Ideally, probiotics used in food and feed production should harbor none of the transferable resistance genes and should be sensitive to pathogen related antibiotics [54]. European Food Safety Authority (EFSA) recommended that bacterial strains harboring virulence factors or transferable ARGs should not be used in animal feeds, probiotic and fermented foods for human [55, 56]. The transmission of ARGs in digestive tracts is a major health concern related to the probiotic application. Unfortunately, until now, ARGs are not included in the standard screening assays before production and application in foods and feeds. Most of the fermentation starter probiotic strains that used for food and animal feed processing were lacking of thorough and rigorous assessments. The donor strain E*fm*4 used in this study was isolated from a retail FSBM product, revealing the risk of dissemination multiple resistant plasmids was not only through the ingestion of food animals, but also via further feed processing like SBM fermentation. In addition to *vanA*, the macrolide-resistant gene *erm* and two aminoglycoside resistant genes *aadK* and *aphA* were also identified in pQZ67 (Fig. 4), explained the resistant of E*fm*4 to erythromycin, kitasamycin and gentamicin. This also indicated that pQZ67 was a multidrug resistance plasmid.

In the past decade, the consumption of FSBM in Asian countries has increased sharply accompanied with the rapid development of animal husbandry [57]. To date, although there has been no report of VRE or other antibiotic-resistance probiotics in FSBM and in the intestines of growing pigs, the potential risk of horizontal transfer of resistance genes is still hanging in cliff, because the consumption is huge while the surveillance is lacked.

## Conclusion

In conclusion, our study illustrated that *vanA* could successfully transfer among enterococci during the fermentation process of soybean meal and also in the digestive tract of growing pigs. Thus, it is suggested that when considering an *Enterococcus* strain as a starter for probiotics, each specific strain should be carefully evaluated to determine the presence of all known virulence factors.

## Additional files

Additional file 1: **Table S1.** Primers used for PCR and DNA sequencing in this study and size of the PCR-targeted products. **Table S2.** Name, abbreviation and drug concentration of 20 antibiotics. (DOCX 19 kb)

Additional file 2: Primers used for partially sequencing of transconjugations and size of the PCR-targeted products. (DOCX 96 kb)

## Abbreviations

ARGs: Antibiotic resistance genes
BEA: Bile Esculin Azide
BHI: Brain heart infusion
CC: Clonal Complex
E*fm*: *Enterococcus faecium*
E*fs*: *Enterococcus faecalis*
FSBM: Fermented soybean meal
MIC: Minimum inhibitory concentration
PCR: Polymerase chain reaction
PFGE: Pulsed-field gel electrophoresis
SBM: Soybean meal
VRE: Vancomycin-resistant enterococci
