# Supplementary material for: Horizontal transfer of vanA between probiotic Enterococcus faecium and Enterococcus faecalis in fermented soybean meal and in digestive tract of growing pigs
Source: J Anim Sci Biotechnol. 2019 Apr 12;10:36. doi: 10.1186/s40104-019-0341-x (PMC6460829; doi:10.1186/s40104-019-0341-x)
Supplement: Supplementary file 1 — Table S1. Primers used for PCR and DNA sequencing in this study and size of the PCR-targeted products. Table S2. Name, abbreviation and drug concentration of 20 antibiotics. (DOCX 19 kb) [file 40104_2019_341_MOESM1_ESM.docx]

**Additional files**

**Additional file 1: Table S1** Primers used for PCR and DNA sequencing in this study and size of the PCR-targeted products.

| Target gene | Primer name | Primer Sequence 5’-3’ | Amplicon length (bp) | Reference |
| --- | --- | --- | --- | --- |
| 16S rDNA | 27-F | AGA GTT TGA TCC TGG CTC AG | 1466 | Liu [2] |
|  | 1492-R | ACG GCT ACC TTG TTA CGA CTT |  |  |
| *E. faecium* | *E.faecium*-F | TTG AGG CAG ACC AGA TTG ACG | 658 | Liu [2] |
|  | *E.faecium*-R | TAT GAC AGC GAC TCC GAT TCC |  |  |
| *E. faecalis* | *E.faecalis*-F | TCA AGT ACA GTT AGT CTT TAT TAG | 941 | Liu [2] |
|  | *E.faecalis*-R | ACG ATT CAA AGC TAA CTG AAT CAG T |  |  |
| *vanA* | *vanA*-F | GGGAAAACGACAATTGC | 732 | Florence et al [57] |
|  | *vanA*-R | GTACAATGCGGCCGTTA |  |  |
| *vanB* | *vanB*-F | CATGATGTGTCGGTAAAATC | 635 | Florence et al [57] |
|  | *vanB-*R | ACCGGGCAGRGTATTGAC |  |  |
| *vanC1* | *vanC1*-F | GATGGCWGTATCCAAGGA | 822 | Florence et al [57] |
|  | *vanC1-*R | GTGATCGTGGCGCTG |  |  |
| *vanC2/C3* | *vanC2/C3*-F | GATGGCWGTATCCAAGGA | 439 | Florence et al [57] |
|  | *vanC2/C3* | ATCGAAAAAGCCGTCTAC |  |  |
| *catA1* | *catA1-*F | CGC CTG ATG AAT GCT CAT CCG | 456 | Kim [27] |
|  | *catA1-*R | CCT GCC ACT CAT CGC AGT AC |  |  |
| *catA2* | *catA2-*F | ATG AAT TTT ACC AGA ATT GAT CTG AA | 639 | Kim [27] |
|  | *catA2-*R | ATT TCA GTA TGT TAT CAC ACA TCA TCT |  |  |
| *catA3* | *catA3-*F | AAA TTG GGT TCG CCG TGA | 1863 | Kim [27] |
|  | *catA3-*R | ATT TAC TGT TAC ACA ACT CTT GTA GCC |  |  |
| *catB* | *catB-*F | TCA AAG GCA AGC TGC TTT CTG AGC | 566 | Kim [27] |
|  | *catB-*R | TAT TAG ACG AGC ACA GCA TGG GCA |  |  |
| *cmlA* | *cmlA-*F | GTT GGC GGT ACT CCC TTG CC | 240 | Kim [27] |
|  | *cmlA-*R | GGC CAC CTC CCA GTA GAA CG |  |  |

**Table S2** Name, abbreviation and drug concentration of 20 antibiotics.

| No. | Catalogue | Name of drug | Abbreviation | Drug concentration |
| --- | --- | --- | --- | --- |
| 1 | Glycopeptides | Vancomycin | VA | 30 μg/disk |
| 2 |  | Teicoplanin | TCL | 30 μg/disk |
| 3 | Rifamycin | Rifampicin | RA | 5 μg/disk |
| 4 | Amphenicols | Chloramphenicol | C | 30 μg/disk |
| 5 | β- lactams | Ampicillin | AM | 10 μg/disk |
| 6 |  | Piperacillin | PIP | 100 μg/disk |
| 7 |  | Cefamedin | CZ | 30 μg/disk |
| 8 |  | Penicillin | P | 10 IU/disk |
| 9 |  | Meropenem | MPN | 10 μg/disk |
| 10 |  | Amoxicillin | AMX | 10 μg/disk |
| 11 | Quinolones | Ofloxacin | OFL | 5 μg/disk |
| 12 |  | Ciprofloxacin | CIP | 5 μg/disk |
| 13 |  | Gatifloxacin | GTF | 5 μg/disk |
| 14 | Aminoglycoside | Gentamicin | GM | 10 μg/disk |
| 15 | Tetracycline | Tetracycline | TE | 30 μg/disk |
| 16 |  | Minocycline | MNO | 30 μg/disk |
| 17 | Macrolides | Erythromycin | E | 15 μg/disk |
| 18 |  | Kitasamycin | GI | 15 μg/disk |
| 19 | Nitrofurans | Nitrofurantoin | FT | 300 μg/disk |
| 20 |  | Furazolidone | FU | 300 μg/disk |
