# Supplementary material for: Horizontal transfer of vanA between probiotic Enterococcus faecium and Enterococcus faecalis in fermented soybean meal and in digestive tract of growing pigs
Source: J Anim Sci Biotechnol. 2019 Apr 12;10:36. doi: 10.1186/s40104-019-0341-x (PMC6460829; doi:10.1186/s40104-019-0341-x)
Supplement: Supplementary file 2 — Primers used for partially sequencing of transconjugations and size of the PCR-targeted products. (DOCX 96 kb) [file 40104_2019_341_MOESM2_ESM.docx]

Supplemental data 1

Primers used for partially sequencing of transconjugations and size of the PCR-targeted products

| Primer name | Primer Sequence 5’-3’ | Amplicon length of transconjugatons (bp) | |
| --- | --- | --- | --- |
|  |  | FSBM | E*fm*4 +E*fs*2 group |
| 0-F | TTG AGT TTC TTC ATG GGT TG | 1322 | 1349 |
| 0-R | GCT GTA TGG ATT ATC TTC TG |  |  |
| 1-F | TAA GAG CGT CTC ATA AGA CT | 1518 | 1519 |
| 1-R | TTC TAG CCT GAC TGA GCA GT |  |  |
| 2-F | GAT GAG TTC TGT CCC CTT TA | 1497 | 1540 |
| 2-R | CAC GAA TAC AAT TGC TAC CA |  |  |
| 3-F | TAC TGC GAA TCC TCT GTG AA | 1496 | 1537 |
| 3-R | TCT GCC TCA TCC TGC TCA CA |  |  |
| 4-F | CGA TGC CAG ACT TGG TTG AT | 1516 | 1524 |
| 4-R | GCA GCC TAC AAA AGG GAT AC |  |  |
| 5-F | AAT CTG CAA TAG AGA TAG CC | 1512 | 1507 |
| 5-R | AAC GGG GAA ATC AAA ATA GC |  |  |
| 6-F | TTG ACC GAA CTG AGA TGA TT | 1440 | 1472 |
| 6-R | CTG ACT TCA CAC TTT CTT GG |  |  |
| 7-F | GAA CCC TTC CAA CTA TAT AG | 1510 | 1486 |
| 7-R | AAT GGG TAC GGT AAA CGA GC |  |  |
| 8-F | TCC ATT TGG CTT GCT TTT GA | 1443 | 1450 |
| 8-R | CCT CTT TTG TCA TCG GTT TA |  |  |
| 9-F | CTA TTA TGT TAA CGG TCC CT | 1531 | 1562 |
| 9-R | GAT TCA TGA TAC CAG AAG AG |  |  |
| 10-F | CTT TGG TTC CCG TCA TTT TC | 1187 | 1160 |
| 10-R | TTT ATG TGC TCC TAG CAT GG |  |  |

For the PCR, the reaction mixture contained 5 μL 10× KOD Buffer (Toyobo, Japan), 5 μL DNTP (Takara, Japan), 3 μL MgSO_4_ (Takara, Japan), 1 μL KOD-NEO-PLUS (Toyobo, Japan), 0.5 μL template DNA, 1 μL upstream primer, 1μL downstream primer and sterile distilled water to bring the final volume to 50 μL.

The reaction was started with a 2 min denaturation step at 98℃. In the PCRs, the temperature cycles consisted of 1 min at 98℃, followed by 1 min at 56℃ and 1 min at 68℃ and each cycle was repeated 30 times. The final cycle was followed by incubation for the reaction mixture for 10 min at 68℃.
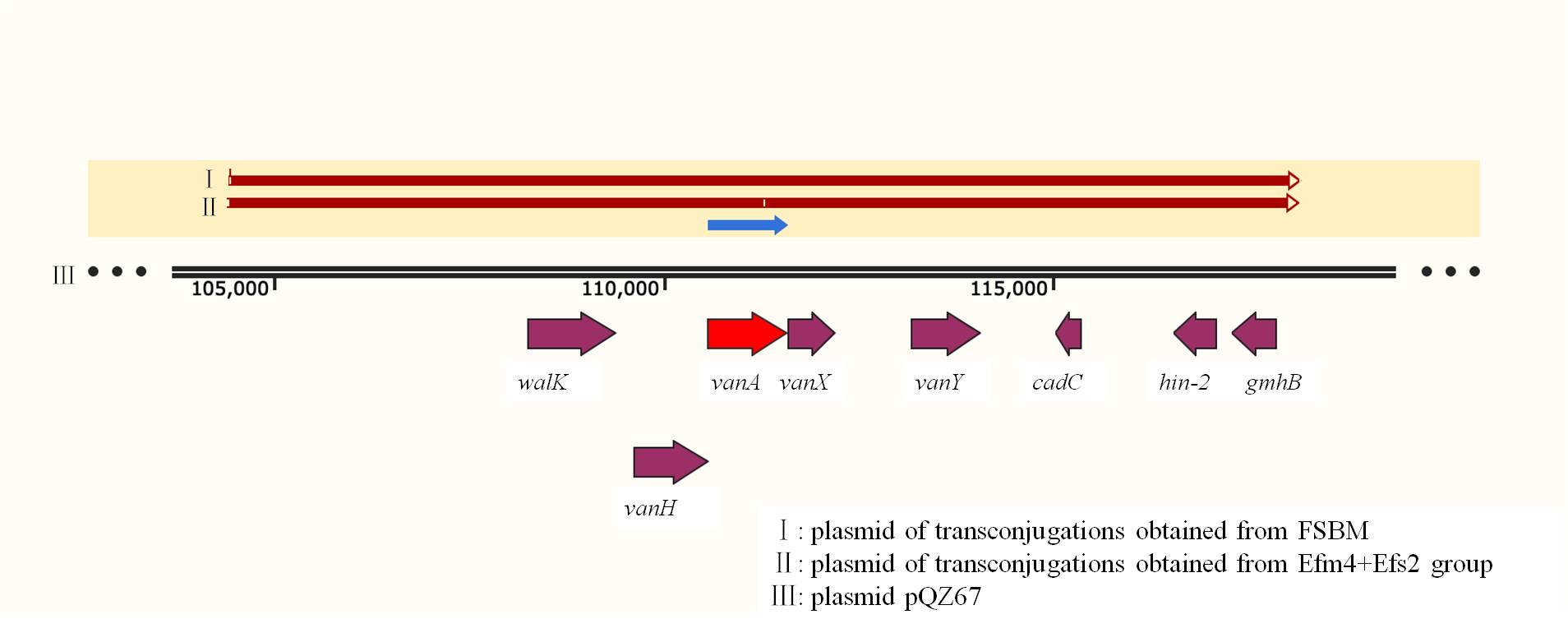


Multiple alignment of plasmids
